# Case report of chest wall reconstruction using biological matrix following resection of a sternal desmoid tumor diagnosed in pregnancy

**DOI:** 10.1093/jscr/rjaf548

**Published:** 2025-08-29

**Authors:** Jamie Walsh, Amber Downes, Rachel Brown, Ross Walsh, Ellen Geary, Christine Quinlan, Donna Eaton

**Affiliations:** Department of Thoracic Surgery, Mater Misericordiae University Hospital Dublin, Eccles St, D07 R2WY, Ireland; Department of Thoracic Surgery, Mater Misericordiae University Hospital Dublin, Eccles St, D07 R2WY, Ireland; Department of Thoracic Surgery, Mater Misericordiae University Hospital Dublin, Eccles St, D07 R2WY, Ireland; Department of Thoracic Surgery, Mater Misericordiae University Hospital Dublin, Eccles St, D07 R2WY, Ireland; Department of Plastic Surgery, Mater Misericordiae University Hospital Dublin, Eccles St, D07 R2WY, Ireland; Department of Plastic Surgery, Mater Misericordiae University Hospital Dublin, Eccles St, D07 R2WY, Ireland; Department of Thoracic Surgery, Mater Misericordiae University Hospital Dublin, Eccles St, D07 R2WY, Ireland

**Keywords:** desmoid tumor, chest wall reconstruction, chest wall resection, collagen, surgical mesh, pregnancy, case report

## Abstract

Desmoid tumors of the chest wall are rare and pose specific challenges in diagnosis, resection and reconstruction. While not known to have potential for metastasis, they have a high risk of recurrence following resection, even with negative margins. Adequate resection has the potential to leave large thoracic defects, the reconstruction of which are technically challenging and often require a multi-disciplinary surgical skill set. We present the case of a 34-year-old woman, diagnosed with desmoid tumor overlying the inferior aspect of her sternum during pregnancy. She underwent resection of the tumor along with the inferior sternum and costal cartilages and subsequent chest wall reconstruction with a biological porcine dermal collagen-based matrix patch (Permacol®) and myocutaneous reconstruction with a transposed, pedicled latissimus dorsi flap. This case demonstrates a novel technique for chest wall reconstruction highlights the complexities of managing these tumors during pregnancy and emphasizes the importance of a multidisciplinary surgical approach.

## Introduction

Desmoid tumors are locally aggressive neoplasms that have no known potential for metastasis but have a high recurrence rate following resection, even with negative margins [1]. They are rare tumors occurring in 2–4/million people per year, with presentation as a chest wall mass, rarer still, accounting for 8% of presentations [2]. This report discusses the case of a 34-year-old woman who presented with an enlarging lesion overlying the xiphisternum, ultimately diagnosed as a desmoid tumor. We describe the use of a novel technique with resection of the tumor along with the inferior sternum and costal cartilages and subsequent chest wall reconstruction with biological porcine dermal collagen-based matrix patch (Permacol®) and myocutaneous reconstruction with a transposed pedicled latissimus dorsi flap.

## Case report

**Figure 1:**
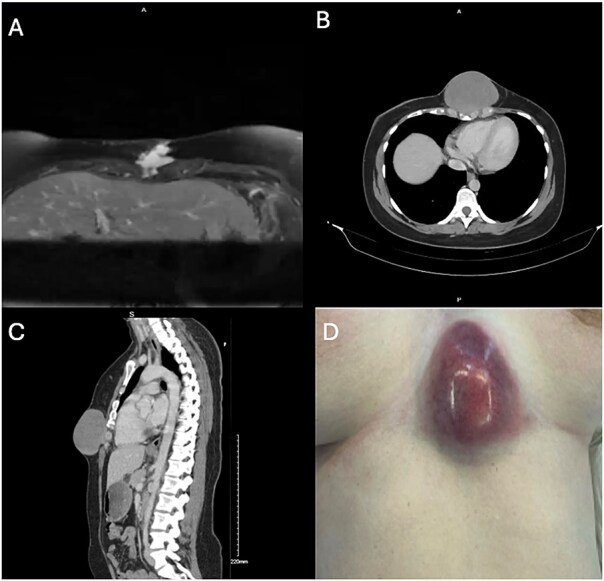
A: MRI axial plane. T2 weighted image. B, C: Non-contrast CT, axial and sagittal planes. D: Photograph of tumor at time of incisional biopsy.

A 34-year-old woman presented with a 3-month history of an enlarging firm lesion over the xiphisternum. There was no history of trauma. MRI demonstrated a 2.2 × 2.5 cm enhancing irregular subcutaneous lesion (Fig. 1A) which on biopsy was suggestive of nodular fasciitis. This was planned for local resection; however, it was deferred as the patient had become pregnant. During her pregnancy, the lesion continued to grow; however, following multidisciplinary discussion, it was felt that the original biopsy was reassuring and likely to represent a benign entity, and thus it was reasonable to continue to wait until after delivery. She had a normal delivery at term. Computed tomography (CT) performed postpartum (Fig. 1B and C) demonstrated an increase in size of the lesion to 8.7 × 8.1 cm. On a physical exam, the lesion had now begun to threaten the overlying skin (Fig. 1D). Given its rapid growth and concern for a more aggressive pathology than original biopsy suggested, an incisional biopsy was taken under general anesthetic instead of outright resection. This revealed a diffuse storiform growth pattern with myxoid stroma, smooth muscle actin positivity, and β-catenin positivity, leading to a diagnosis of a desmoid tumor.

**Figure 2:**
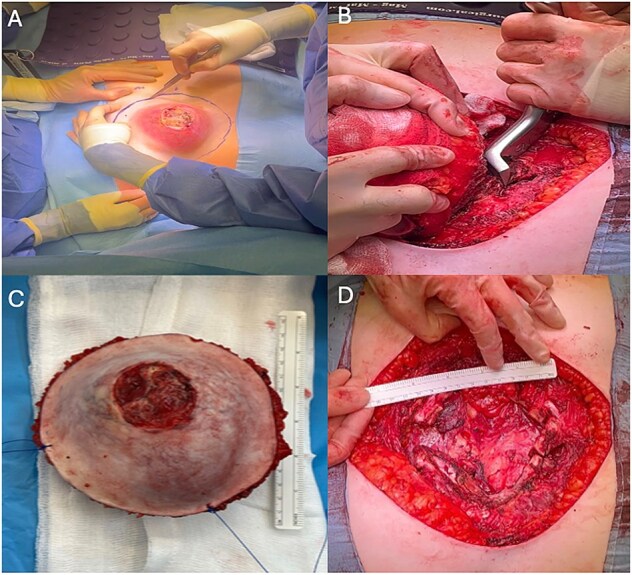
A: Incision around tumor with wide margin. B: Costal resection. C: Resected specimen. D: Large (14 × 12 cm) anterior thoracic defect.

The patient underwent a combined thoracic and plastic surgery case with chest wall resection, including the inferior sternum and costal cartilage, followed by reconstruction with Permacol mesh and a pedicled latissimus dorsi musculocutaneous flap. An incision was made around the tumor with wide margins, with dissection down to the chest wall (Fig. 2A). The intercostal spaces were entered and the costal cartilage resected circumferentially around the inferior sternum. A margin of costal cartilage was left intact bilaterally to avoid disrupting continuity of the thoracic cage (Fig. 2B). A transverse sternotomy was then performed and the tumor was removed en bloc along with the inferior sternum (Fig. 2C).

**Figure 3:**
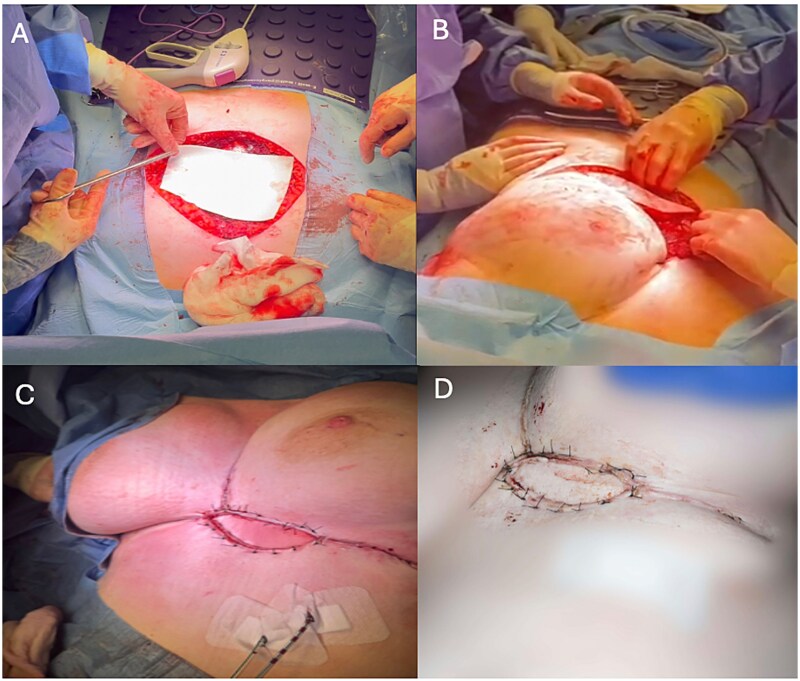
A: Chest wall reconstruction with Permacol mesh. B: Tunneled pedicled latissimus dorsi flap. C: Closure. D: Wound reviewed at ten days post-operatively.

Resection left a 14 × 12 cm defect in the inferior chest wall (Fig. 2D). First, the chest wall was reconstructed using a 20 cm^2^ acellular porcine dermal collagen-based matrix patch (Permacol® Tissue Science Laboratory, Covington, USA). This was cut to size and secured to the sternum with 2–0 continuous Prolene sutures. (Fig. 3A). A pedicled latissimus dorsi myocutaneous flap was then raised to cover the patch. A prepectoral plane was dissected to create a left submammary tunnel, and the flap transposed anteriorly and positioned free of tension (Fig. 3B). Four Redivac drains were placed before closure of muscles and skin (Fig. 3C).

Post-operatively, the patient’s pain was managed with a thoracic epidural, fentanyl patient-controlled analgesia, and multimodal oral analgesia. There were no postoperative complications, and she was discharged home on postoperative day 12. The wounds have healed well and final histology confirmed the diagnosis of a desmoid tumor with an R0 resection (Fig. 3D).

## Discussion

Desmoid tumors are rare tumors, and presentation as chest wall mass is rarer still [1]. A subset are estrogen-sensitive tumors and associated with pregnancy, as in this case, which poses unique challenges in management and, particularly, the timing of resection [2]. While not possessing any known metastatic potential, they are locally aggressive and infiltrative tumors with high recurrence rates following resection, even with negative margins. [3] The largest study to date found a recurrence rate of ~18% even with negative margins [3]. Tumors of the chest wall pose particular challenges for resection and reconstruction. Traditionally, synthetic mesh or bars have been used for chest wall reconstruction, but here we present the use of a biological matrix to repair the large defect left from resection [4]. This, combined with a tunneled, pedicled latissimus dorsi flap, yielded excellent cosmetic results for the patient with minimal morbidity and a good oncological outcome.

## Conclusion

This case highlights the importance of a multidisciplinary approach in the management of complex surgical cases. The delay in surgical intervention due to pregnancy and the subsequent change in diagnosis from nodular fasciitis to desmoid tumor meant a technically more challenging surgical resection was required, albeit with an excellent cosmetic and oncological outcome.
